# Efficacy and safety of bone marrow mononuclear cell therapy for myocardial infarction: a dose–response meta-analysis

**DOI:** 10.3389/fcell.2026.1952788

**Published:** 2026-10-09

**Authors:** Jianzhou Tian, Jianing Wang, Ziheng Cui, Cui Xie, Pei Qiao, Jun Shen

**Affiliations:** Department of Cardiology, Arteriosclerosis Cardiovascular Disease Clinical Medical Research Center of Hubei Province, Renmin Hospital, Hubei University of Medicine, Shiyan, China

**Keywords:** bone marrow mononuclear cells, dose–response, meta-analysis, myocardial infarction, randomized controlled trial

## Abstract

**Background:**

Bone marrow mononuclear cells (BMMNCs) have been investigated as a potential regenerative therapy for myocardial infarction (MI). However, whether their clinical benefits are sustained over time and what constitutes an optimal therapeutic dose remain uncertain. This meta-analysis aimed to evaluate the efficacy, safety, and dose–response relationship of BMMNC therapy in patients with MI.

**Methods:**

Randomized controlled trials (RCTs) comparing BMMNC therapy with standard therapy or placebo in patients with MI were systematically searched in relevant databases. Statistical analyses were performed using R. Continuous outcomes were summarized as mean differences (MDs) with 95% confidence intervals (CIs), and dichotomous outcomes were summarized as risk ratios (RRs) with 95%CIs. Random-effects models were used for pooled analyses. Subgroup analyses, sensitivity analyses, meta-regression, and restricted cubic spline models were performed to explore the effects of follow-up duration and cell dose on treatment outcomes.

**Results:**

A total of 43 RCTs involving 2,213 patients receiving BMMNC therapy and 1,686 control patients were included. BMMNC therapy significantly improved left ventricular ejection fraction (LVEF) at 6 months (MD = 2.57%, 95%CI: 0.79 to 4.35; p = 0.007), but this effect was attenuated at 12 months and did not reach statistical significance (MD = 1.59%, 95%CI: −0.06 to 3.23; p = 0.057). At the final follow-up, BMMNC therapy was still associated with a significant improvement in LVEF (MD = 2.24%, 95%CI: 0.77 to 3.71; p = 0.004). Meta-regression showed that the improvement in LVEF decreased significantly with longer follow-up duration (β = −0.296 per month, 95%CI: −0.549 to −0.043; p = 0.024). No significant linear or nonlinear dose–response relationship was observed between cell dose and LVEF improvement. Safety analysis showed that BMMNC therapy significantly reduced the risk of major adverse cardiac events (MACE) overall (RR = 0.62, 95%CI: 0.45 to 0.86; p = 0.0036). However, this benefit was mainly observed in low- and intermediate-dose studies (<10^9^ cells), whereas high-dose studies (>10^9^ cells) did not show a clear benefit (RR = 1.53, 95%CI: 0.40–5.82), suggesting a potential threshold in the dose–safety relationship. Further meta-regression indicated that increasing cell dose was significantly associated with a higher risk of MACE (β = 0.341, 95%CI: 0.104 to 0.578; p = 0.0062). Subgroup analyses suggested that a dose range of 10^8^ to 10^9^ cells may provide a favorable balance between LVEF improvement and safety.

**Conclusion:**

BMMNC therapy may improve LVEF in the short term and reduce the risk of MACE in patients with MI; however, the LVEF benefit appears to diminish over time. Current evidence does not support a significant linear or nonlinear dose–response relationship between cell dose and LVEF improvement, although higher cell doses may be associated with increased MACE risk. Taken together, 10^8^ to 10^9^ cells may represent a potentially appropriate therapeutic dose range, although this finding requires confirmation in further high-quality RCTs with longer follow-up and standardized cell-processing protocols.

## Introduction

Myocardial infarction (MI) remains one of the leading causes of cardiovascular mortality and disability worldwide (Martin et al., 2025). Although early reperfusion therapy and evidence-based pharmacological treatment have substantially improved short-term outcomes in patients with acute MI, irreversible cardiomyocyte loss, adverse left ventricular remodeling, and subsequent deterioration of cardiac function remain common after infarction (Rao et al., 2025; Ozaki et al., 2026). These changes contribute to an increased risk of heart failure and long-term adverse cardiovascular events (Yao et al., 2026). Therefore, therapeutic strategies that can promote myocardial repair, improve cardiac function, and optimize long-term prognosis in addition to standard care remain an important unmet clinical need (Udell et al., 2025).

With the development of regenerative medicine, cell-based therapy has emerged as a potential approach to enhance post-infarction cardiac repair (Wan et al., 2025). Among various candidate cell types, bone marrow mononuclear cells (BMMNCs) have been among the most widely investigated in clinical studies of MI because they are relatively easy to obtain, feasible for autologous use, and were introduced into clinical research at an early stage (Uemura et al., 2022). BMMNCs represent a heterogeneous cell population that typically includes hematopoietic progenitor cells, endothelial progenitor cells, and other mononuclear cell components. Previous studies have suggested that BMMNCs may contribute to myocardial repair mainly through paracrine mechanisms, including promotion of angiogenesis, modulation of inflammatory responses, inhibition of apoptosis, and improvement of the local reparative microenvironment (Yoon et al., 2010; You et al., 2024; Zhao et al., 2026).

However, the clinical value of BMMNC therapy for MI remains controversial. Over the past 2 decades, multiple randomized controlled trials (RCTs) have evaluated the efficacy of BMMNC therapy, but their findings have been inconsistent (Mathur et al., 2020; Lee et al., 2024; Mahram et al., 2025). Some trials reported modest improvements in left ventricular ejection fraction (LVEF) and potential favorable effects on left ventricular remodeling (Wollert et al., 2004; Schächi et al., 2006), while others failed to demonstrate clear clinical benefit (Mathur et al., 2020; Sürder et al., 2013; Traverse et al., 2011). Previous meta-analyses have also reached inconsistent conclusions, suggesting that the overall treatment effect may be limited and that the durability of long-term benefit remains uncertain (Lee et al., 2024; Attar et al., 2022; Moeswir et al., 2025). These discrepancies indicate that cell dose and follow-up duration may be important factors influencing the therapeutic effect of BMMNCs.

Among these factors, the administered cell dose is of particular interest because it may influence both efficacy and safety, yet the optimal dose range remains unclear (Yan et al., 2024; Rettkowski et al., 2025; Karim and Tompkins, 2025). Most previous RCTs primarily focused on whether BMMNC therapy was effective, while systematic evidence regarding differences in efficacy and safety across dose levels remains limited. In addition, major adverse cardiac events (MACE) represent a clinically important endpoint for assessing the overall risk–benefit profile of BMMNC therapy in MI (Lee et al., 2024; Mahram et al., 2025; Hosseinpour et al., 2023).

Therefore, this study systematically included RCTs comparing BMMNC therapy with control treatment in patients with MI. We performed a meta-analysis to evaluate the effects of BMMNC therapy on cardiac function and safety outcomes, and further investigated dose–response relationships between cell dose and treatment effects. We also examined changes in treatment effects across different follow-up durations. Compared with previous studies, the key contribution of this study is that it systematically quantified the dose–response relationship between cell dose and MACE risk, while simultaneously incorporating follow-up duration and dose into a meta-regression framework.

## Methods

### Study protocol and registration

The design, conduct, and reporting of this study followed the Preferred Reporting Items for Systematic Reviews and Meta-Analyses (PRISMA) 2020 statement (Page et al., 2021). The study protocol was prospectively registered in PROSPERO under the registration number CRD420261378197.

### Literature search strategy

A systematic literature search was conducted in PubMed, Embase, the Cochrane Library, Web of Science, Scopus, and ClinicalTrials.gov to identify RCTs comparing BMMNC therapy with standard therapy or placebo in patients with MI. Grey literature was also searched, including Google Scholar, relevant reviews, and the reference lists of included studies. The search was restricted to English-language publications and covered the period from database inception to 3 December 2025. The search strategy combined controlled vocabulary terms and free-text terms, including but not limited to “myocardial infarction”, “acute myocardial infarction”, “STEMI”, “bone marrow mononuclear cells”, and “bone marrow-derived mononuclear cells”. The detailed search strategy is provided in Supplementary Material S1.

### Inclusion and exclusion criteria

Inclusion criteria: Studies were eligible for inclusion if they met all of the following criteria: (1) the study population consisted of adult patients with a definite diagnosis of MI; (2) the intervention consisted of BMMNC therapy in addition to standard care, administered via intracoronary infusion, intramyocardial injection, or intravenous infusion; (3) the control group received placebo plus standard care or standard care alone, with standard care defined as guideline-recommended baseline therapy, including reperfusion therapy and conventional pharmacological treatment (Martin et al., 2025; Rao et al., 2025); (4) the study design was an RCT; (5) the study reported at least one efficacy or safety outcome of interest, including LVEF or MACE, with MACE including cardiovascular death, recurrent MI, stroke, or related events; and (6) the study clearly reported the total number of infused BMMNCs or provided sufficient information to estimate the administered cell dose.

Exclusion criteria: Studies were excluded if they met any of the following criteria: (1) reviews, systematic reviews, meta-analyses, editorials, letters, case reports, animal studies, or *in vitro* studies; (2) duplicate publications or multiple reports from the same study cohort, in which case the report with the most complete information or the longest follow-up was retained; (3) studies without extractable effect estimates or lacking essential raw data for quantitative synthesis; (4) conference abstracts without sufficient data for analysis; or (5) non-English publications.

Although freshly isolated BMMNC preparations may contain rare mesenchymal stromal progenitor cells, they are distinct from selected or culture-expanded bone marrow-derived mesenchymal stromal/stem cell products; therefore, trials evaluating the latter alone were excluded from this meta-analysis.

### Study selection and data extraction

Two investigators independently screened the literature and extracted data, and the extracted information was cross-checked. Any disagreements were resolved by discussion with a third investigator. Extracted data included first author, publication year, country, sample size, participant age, timing of cell administration, total number of infused cells, follow-up duration, and outcome data. For trials with multiple intervention arms, each eligible intervention arm was extracted separately. For studies reporting multiple follow-up time points, data were extracted according to predefined time points, including 6 months, 12 months, and final follow-up.

### Risk-of-bias assessment

The methodological quality of included RCTs was independently assessed by two investigators using the revised Cochrane risk-of-bias tool for randomized trials (RoB 2.0) (Sterne et al., 2019). The evaluated domains included bias arising from the randomization process, bias due to deviations from intended interventions, bias due to missing outcome data, bias in measurement of outcomes, and bias in selection of the reported result. Each domain was judged as “low risk”, “some concerns”, or “high risk”. Disagreements were resolved through discussion.

### Statistical analysis

All statistical analyses were performed using R software, mainly with the meta, metafor, and rms packages. Continuous outcomes were summarized as MDs with 95%CIs, and dichotomous outcomes were summarized as RRs with 95%CIs. Between-study heterogeneity was assessed using Cochran’s Q test and quantified using the I^2^ statistic (Lortie and Filazzola, 2020; Shim and Lee, 2019). Given the expected clinical and methodological heterogeneity across included studies in terms of patient populations, treatment regimens, and follow-up durations, random-effects models were used for all meta-analyses. Stratified meta-analyses were conducted according to predefined follow-up time points, including 6 months, 12 months, and final follow-up. Leave-one-out sensitivity analyses were performed to assess the robustness of pooled estimates.

### Meta-regression and dose–response analysis

To explore potential sources of between-study heterogeneity, meta-regression analyses were performed to assess the effects of follow-up duration and infused BMMNC dose on treatment outcomes. When the original study directly reported the mean or median cell dose, that value was used. When only a dose range was reported, the midpoint of the range was used as the representative dose. Cell dose was first log_10_-transformed and entered as a covariate in a linear model to evaluate the linear relationship between cell dose and treatment effect. Restricted cubic spline models with three knots were then used to examine potential nonlinear dose–response relationships and to assess the validity of the linear assumption (Shim and Lee, 2019). Dose categories (<10^8^, 10^8^–10^9^, and >10^9^ cells) were prespecified based on dose ranges commonly used in previous RCTs and clinically relevant orders of magnitude.

### Publication bias

When at least 10 studies were available for a given outcome, funnel plots were used for visual assessment of publication bias, and Egger’s test was performed to evaluate small-study effects.

## Results

### Literature search results

A total of 41,291 records were identified through the literature search. After duplicate removal, title and abstract screening, and full-text assessment, 43 studies (Mathur et al., 2020; Wollert et al., 2004; Schächi et al., 2006; Traverse et al., 2011; Ge et al., 2006; Huikuri et al., 2008; Janssens et al., 2006; Jazi et al., 2012; Kaminek et al., 2010; Lipiec et al., 2009; Nicolau et al., 2018; Nog et al., 2009; Plewka et al., 2009; Quyyumi et al., 2011; Schaefer et al., 2010; Suárez et al., 2007; Beitnes et al., 2009; Cao et al., 2009; Choudry et al., 2016; Colombo et al., 2011; Dill et al., 2009; Grajek et al., 2010; Kaminek et al., 2008; Laguna et al., 2018; Lamirault et al., 2017; San et al., 2015; Yao et al., 2009; Meyer et al., 2009; Skalicka et al., 2012; Traverse et al., 2018; Wöhrle et al., 2013; Nair et al., 2015; Tendera et al., 2009; Wöhrle et al., 2010; Meluzín et al., 2008; Piepoli et al., 2010; Quyyumi et al., 2017; Schächinger et al., 2006; Sürder et al., 2016; Assmus et al., 2010; Meyer et al., 2006; Traverse et al., 2012; Naseri et al., 2018) met the eligibility criteria and were included in the final analysis. The study selection process is shown in Figure 1.

**FIGURE 1:**
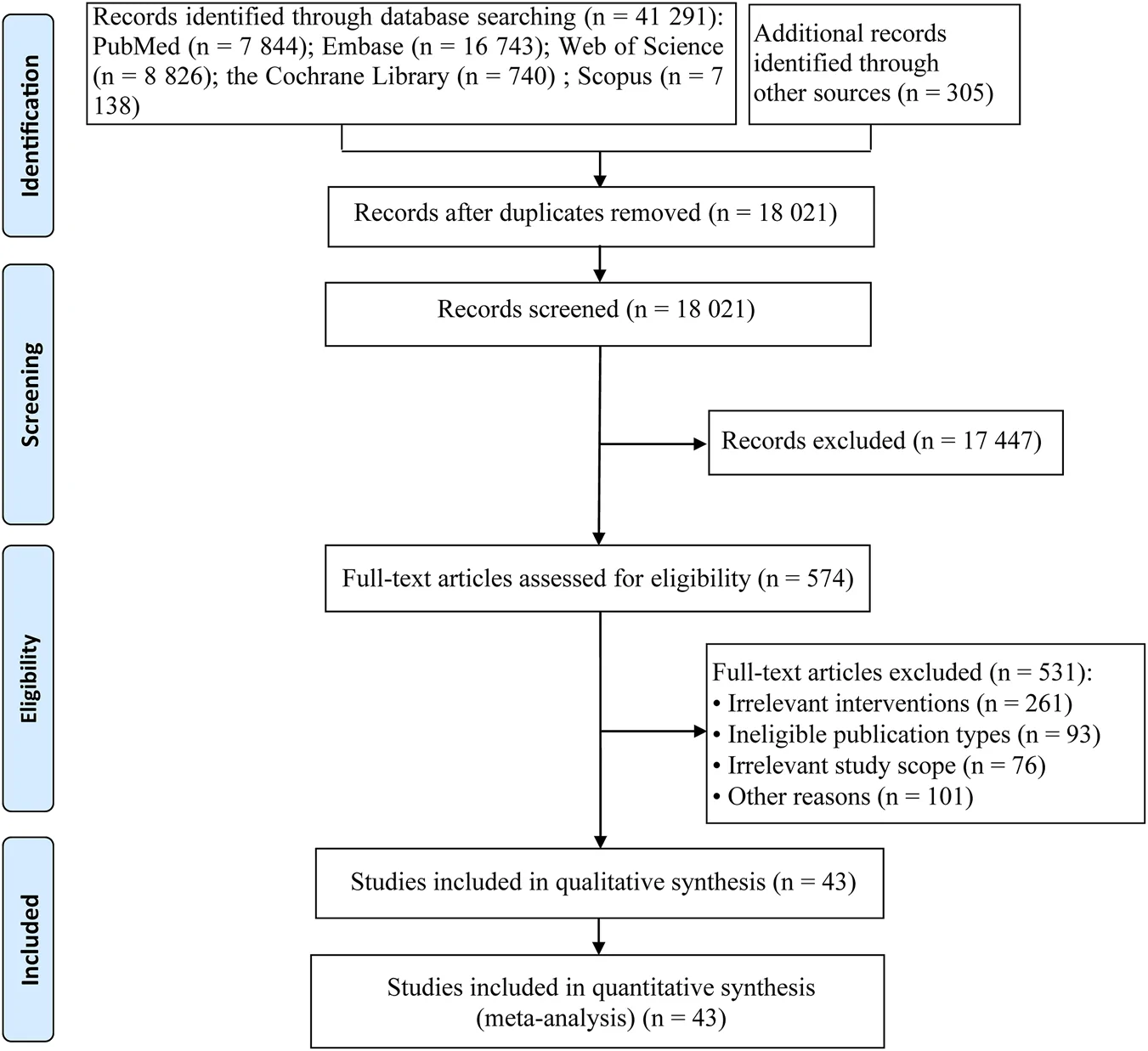
Study selection flow diagram. PRISMA flow diagram showing the process of literature search, screening, eligibility assessment, and final inclusion of studies in the meta-analysis.

### Study characteristics

The 43 included studies enrolled 2,213 patients receiving BMMNC therapy and 1,686 control patients. Among them, 29 studies evaluated LVEF outcomes and 32 studies reported MACE outcomes. The main characteristics of the included studies are summarized in Table 1. Of the 43 included studies, 42 delivered BMMNCs via the intracoronary route and one via the intramyocardial route; none used intravenous administration.

**TABLE 1:** Summary of the main characteristics of the included studies.

Author	Year	Country	Age(years)		Sample size		Male/Female		Cell dose (10^7^)	Delivery route	Follow-up time(months)	Outcome
			Treatment	Control	Treatment	Control	Treatment	Control				
(Ge et al., 2006)	2006	China	58 ± 11	59 ± 8	10	10	8/2	10/0	4	IC	6	LVEF
(Huikuri et al., 2008)	2008	Finland	60 ± 10	59 ± 10	40	40	36/4	34/6	40.2	IC	6	LVEF, MACE
(Janssens et al., 2006)	2006	Belgium	55.8 ± 11	57.9 ± 10	33	34	27/6	28/6	17.2	IC	6	LVEF
(Jazi et al., 2012)	2012	Iran	48 ± 2.5	45.2 ± 3.2	16	16	11/5	14/2	246	IC	6	LVEF, MACE
(Kaminek et al., 2010)	2008	Czech	55 ± 8	54 ± 8	31	31	26/5	28/3	10	IC	6	LVEF
(Lipiec et al., 2009)	2009	Poland	57 ± 9	59 ± 9	26	10	18/8	7/3	0.369	IC	6	LVEF
(Nicolau et al., 2018)	2018	Brazil	59.2 ± 9.4	58.7 ± 9.3	66	55	53/13	45/11	10	IC	6	LVEF
(Nog et al., 2009)	2009	Brazil	59.7 ± 14.3	57.2 ± 10.8	14	6	10/4	4/2	10	IC	6	LVEF
	​	​	53.6 ± 8.3	​	10	​	7/3	​				
(Plewka et al., 2009)	2009	Poland	56 ± 9	56 ± 8	38	18	26/12	14/4	14.4	IC	6	LVEF, MACE
(Quyyumi et al., 2011)	2011	United States of America	52	52	15	15	14/1	13/2	1	IC	6	LVEF, MACE
(Schächi et al., 2006)	2006a	Germany	55 ± 11	57 ± 11	101	103	83/18	84/19	23.6	IC	6	LVEF, MACE
(Schaefer et al., 2010)	2010	Germany	53 ± 14.8	59.2 ± 13.5	28	28	19/9	22/6	2500	IC	6	LVEF
(Suárez et al., 2007)	2007	Spain	52 ± 12	55 ± 11	10	10	8/2	7/3	90	IC	6	LVEF
(Traverse et al., 2011)	2011	United States of America	57.6 ± 11	54.6 ± 11	58	29	46/12	26/3	14.7	IC	6	LVEF, MACE
(Beitnes et al., 2009)	2009	Norway	58.1 ± 9.6	56.7 ± 9.6	50	50	42/8	42/8	6.8	IC	6/12/36	LVEF, MACE
(Cao et al., 2009)	2009	United States of America	50.7 ± 1.1	51.0 ± 1.0	41	45	39/2	42/3	12.5	IC	6/12/48	LVEF, MACE
(Choudry et al., 2016)	2016	United Kingdom	56.4 ± 10.4	56.7 ± 10.7	55	45	46/9	41/4	5.98	IC	12	LVEF, MACE
(Colombo et al., 2011)	2011	Italy	53.8 ± 3.7	53.5 ± 3.9	5	5	5/0	5/0	0.92	IC	12	LVEF, MACE
	​	​	48.8 ± 9.5	​	5	​	4/1	​				
(Dill et al., 2009)	2009	Germany	57.9 ± 10.7	54.6 ± 11.4	27	27	24/3	25/2	0.1	IC	6/12	LVEF
(Grajek et al., 2010)	2010	Poland	49.9 ± 8.4	50.9 ± 9.3	31	14	27/4	12/2	234	IC	6/12	LVEF, MACE
(Kaminek et al., 2008)	2010	Czech	54 ± 9	56 ± 9	37	36	32/5	32/4	10	IC	6/12	LVEF
(Laguna et al., 2018)	2018	Spain	62.63 ± 8.35	64.8 ± 11.5	8	9	7/1	8/1	1	IM	12	LVEF
(Lamirault et al., 2017)	2017	France	56 ± 12	55 ± 11	52	49	42/10	44/5	9.8	IC	12	LVEF, MACE
(San et al., 2015)	2015	Spain	54 ± 11	57 ± 11	30	31	29/1	28/2	8.3	IC	12	LVEF, MACE
	​	​	57 ± 9	​	30	​	25/5	​				
	​	​	56 ± 8	​	29	​	25/4	​				
(Yao et al., 2009)	2009	China	52.1 ± 6.3	52.7 ± 7.8	12	12	10/2	11/1	19	IC	6/12	LVEF, MACE
​	​	​	51.3 ± 7.4	​	15	​	12/3	​				
(Meyer et al., 2009)	2009	Germany	53 ± 14.8	59.2 ± 13.5	30	30	20/10	22/8	2500	IC	18/24	LVEF
(Skalicka et al., 2012)	2012	Czech	61 ± 14	54 ± 10	17	10	12/5	10/0	264	IC	6/24	LVEF, MACE
(Traverse et al., 2018)	2018	United States of America	55.9 ± 11.0	56.4 ± 10.4	58	27	51/7	23/4	15	IC	6/12/24	LVEF, MACE
(Wöhrle et al., 2013)	2013	Germany	61.0 ± 8.1	61.1 ± 9.3	29	13	26/1	8/5	32.4	IC	6/12/24/36	LVEF
(Nair et al., 2015)	2015	India	48.1 ± 9.7	49.0 ± 9.8	125	125	111/14	109/16	55.8	IC	6	MACE
(Tendera et al., 2009)	2009	Poland	55	59	80	40	56/24	30/10	17.8	IC	6	MACE
	​	​	58	​	80	​	51/29	​				
(Wöhrle et al., 2010)	2010	Germany	61 ± 8.1	61.1 ± 9.3	29	13	26/3	8/5	38.1	IC	6	MACE
(Wollert et al., 2004)	2004	Germany	53 ± 14.8	59.2 ± 13.5	30	30	20/10	22/8	2500	IC	6	MACE
(Meluzín et al., 2008)	2008	Czech	54 ± 2	55 ± 2	20	20	19/1	18/2	1	IC	6/12	MACE
	​	​	54 ± 2	​	20	​	18/2	​	10			
(Piepoli et al., 2010)	2010	Italy	63.1 ± 2.4	67.0 ± 2.7	19	19	13/6	13/6	41.8	IC	12	MACE
(Quyyumi et al., 2017)	2017	United States of America	57.1 ± 10.1	56.4 ± 10.1	78	83	66/12	66/17	1.49	IC	6/12/24	MACE
(Schächinger et al., 2006)	2006b	Germany	55 ± 11	57 ± 11	101	103	83/18	84/19	0.1	IC	12	MACE
(Sürder et al., 2016)	2016	Switzerland	55 ± 15	56 ± 14.5	65	55	56/9	46/9	27.5	IC	6/12	MACE
	​	​	62 ± 15	​	63	​	52/11	​	​	​	​	
(Assmus et al., 2010)	2010	Germany	55 ± 11	57 ± 11	101	103	83/18	84/19	0.1	IC	24	MACE
(Mathur et al., 2020)	2020	European	59 ± 11	60 ± 11	185	190	155/30	147/43	14	IC	24	MACE
(Meyer et al., 2006)	2006	Germany	53 ± 14.8	59.2 ± 13.5	30	30	20/10	22/8	2500	IC	18/24	MACE
(Traverse et al., 2012)	2012	United States of America	56.9 ± 11.1	57 ± 10.2	79	41	69/10	36/5	15	IC	6	MACE
(Naseri et al., 2018)	2018	Iran	51.5 ± 7.5	55.5 ± 8.6	51	26	46/5	23/3	56.463	IC	18/24	MACE

LVEF, left ventricular ejection fraction; MACE, major adverse cardiac events; IC, intracoronary delivery; IM, intramyocardial delivery.

### Risk-of-bias assessment

According to the RoB 2.0 assessment, 10 studies were judged to have a low risk of bias, 20 studies raised some concerns, and 13 studies were judged to have a high risk of bias. The results of the risk-of-bias assessment are presented in Supplementary Figure S1.

### Meta-analysis results

#### Left ventricular ejection fraction at 6 months

A total of 22 RCTs reported LVEF at 6 months, including 744 patients in the BMMNC group and 581 patients in the control group. Random-effects meta-analysis showed that BMMNC therapy significantly improved LVEF at 6 months compared with control treatment (MD = 2.57%, 95%CI: 0.79 to 4.35; I^2^ = 50.6%). The corresponding forest plot is shown in Figure 2.

**FIGURE 2:**
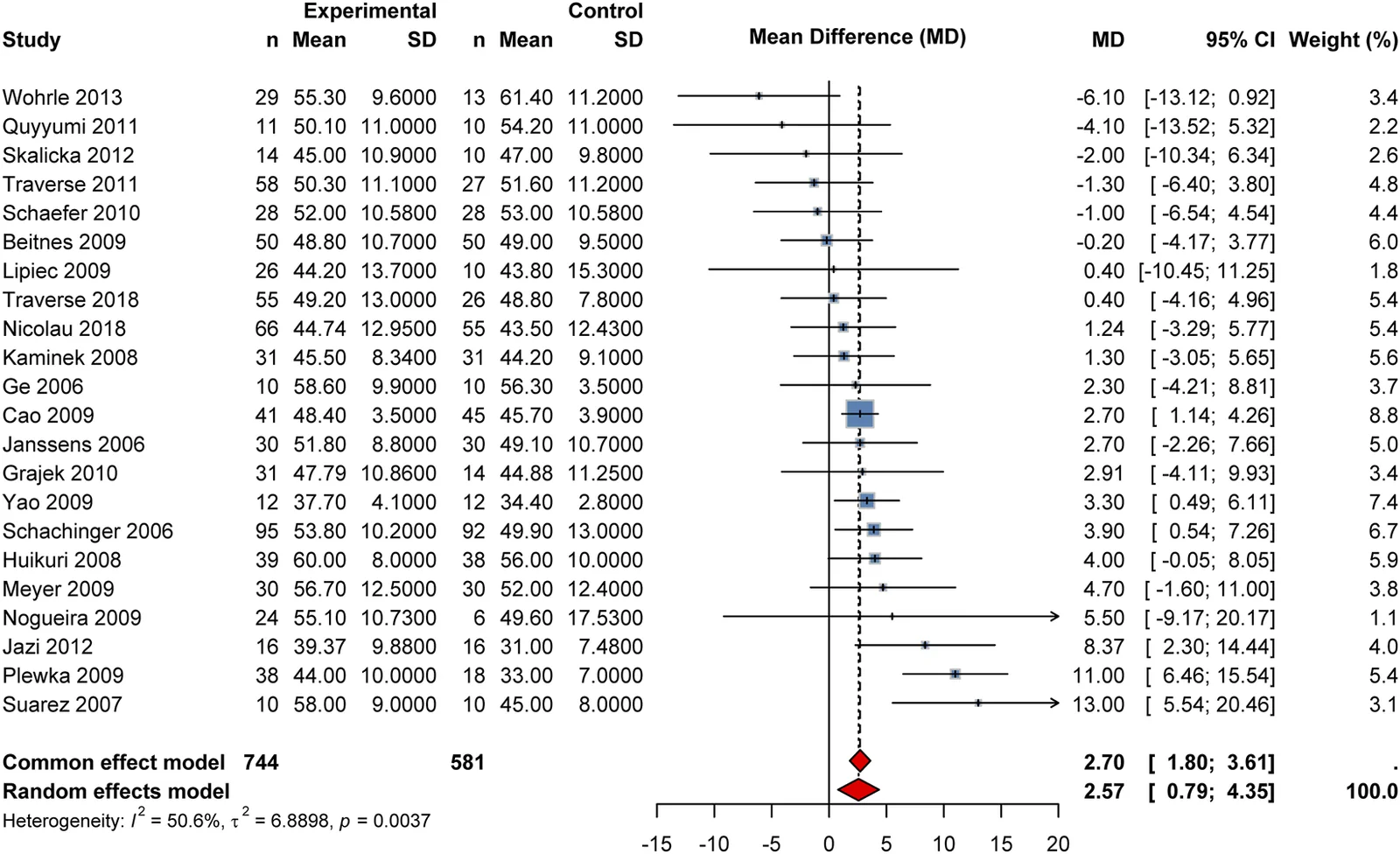
Forest plot of LVEF at 6 months. Pooled mean difference in left ventricular ejection fraction (LVEF) between BMMNC therapy and control groups at 6-month follow-up.

Dose subgroup analysis showed that the intermediate-dose group (10^8^–10^9^ cells; 12 studies) had a significant improvement in LVEF (MD = 2.56%, 95%CI: 0.24–4.88), whereas the low-dose group (<10^8^ cells; four studies) and high-dose group (>10^9^ cells; six studies) did not show statistically significant benefits. No significant between-subgroup difference was observed. The subgroup forest plot is shown in Supplementary Figure S2A. Dose–response meta-regression did not identify a significant linear or nonlinear relationship between cell dose and 6-month LVEF improvement (β = 0.803, p = 0.472; Figure 3A). Sensitivity analysis indicated that the pooled result was robust (Supplementary Figure S3A). Egger’s test did not suggest significant publication bias (p = 0.732; Supplementary Figure S4A).

**FIGURE 3:**
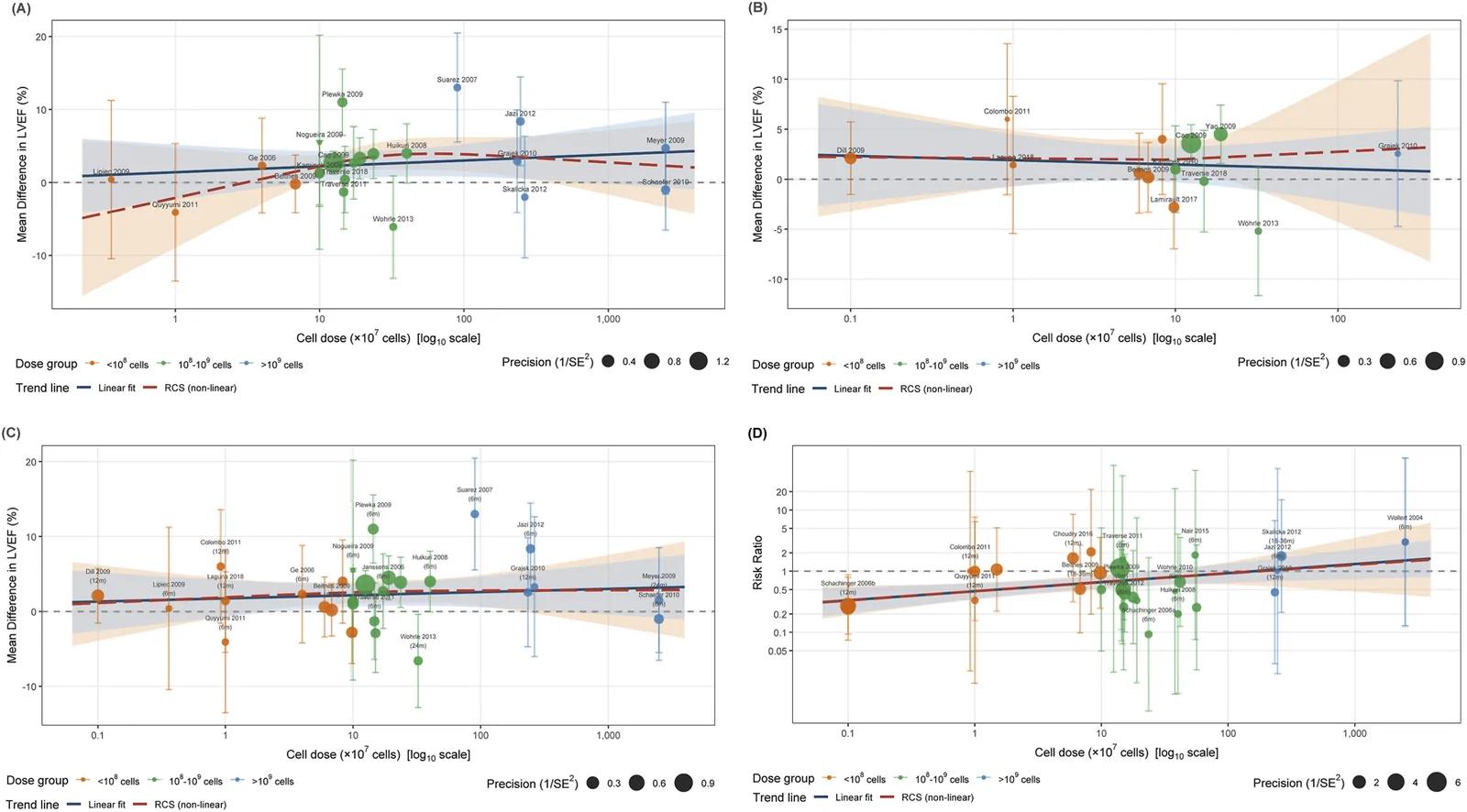
Dose-response analyses of BMMNC therapy. Dose-response relationships between administered BMMNC dose and treatment effects on LVEF at 6 months **(A)**, LVEF at 12 months **(B)**, overall LVEF **(C)**, and MACE risk **(D)**. Cell dose is shown on a log10 scale. Bubble size represents study precision. Solid lines indicate linear fits, and dashed lines indicate restricted cubic spline fits.

#### Left ventricular ejection fraction at 12 months

A total of 13 RCTs reported LVEF at 12 months, including 417 patients in the BMMNC group and 341 patients in the control group. Random-effects meta-analysis showed that the improvement in LVEF at 12 months did not reach statistical significance compared with control treatment (MD = 1.59%, 95%CI: −0.06 to 3.23; I^2^ = 38.9%). The corresponding forest plot is shown in Figure 4.

**FIGURE 4:**
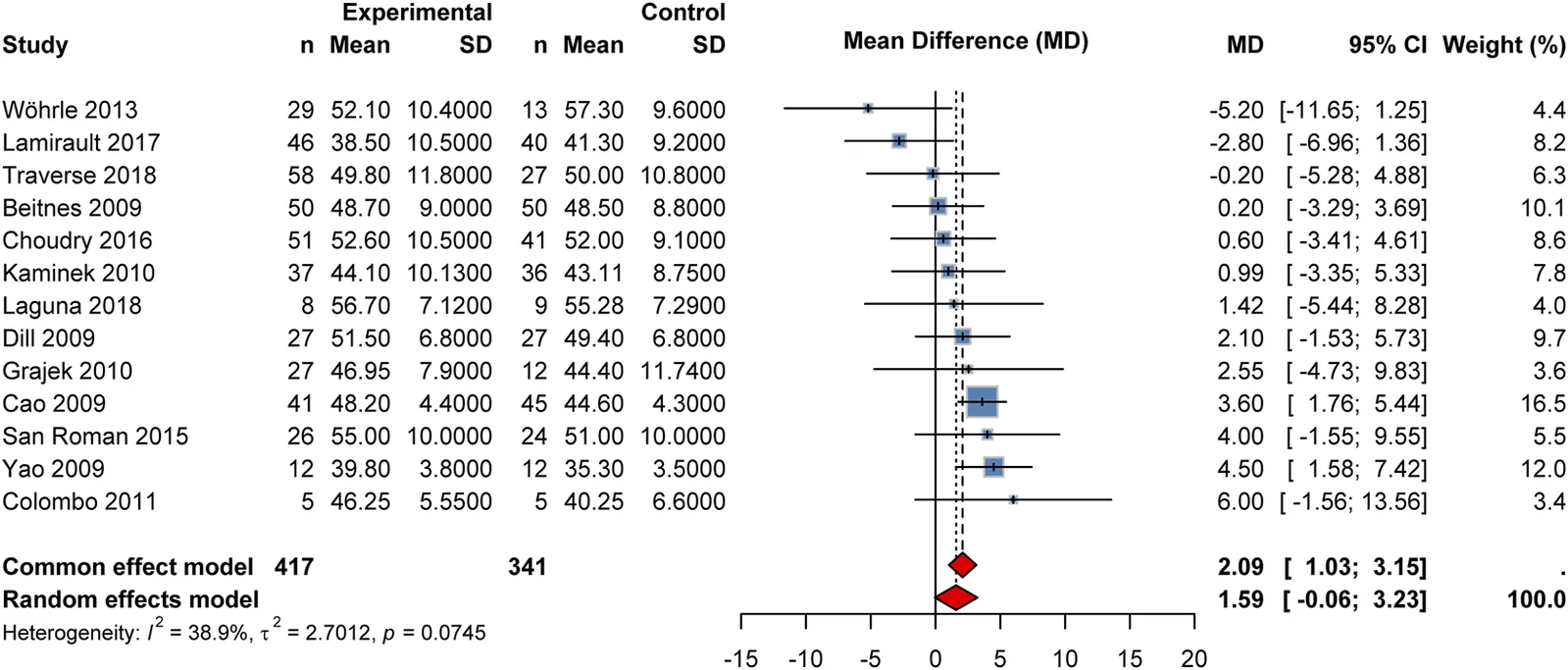
Forest plot of LVEF at 12 months. Pooled mean difference in LVEF between BMMNC therapy and control groups at 12-month follow-up.

Dose subgroup analysis showed that none of the low-dose group (<10^8^ cells; seven studies), intermediate-dose group (10^8^–10^9^ cells; five studies), or high-dose group (>10^9^ cells; one study) demonstrated a statistically significant benefit. The between-subgroup difference was not significant (p = 0.803), and the subgroup forest plot is shown in Supplementary Figure S2B. Dose–response meta-regression did not identify a significant linear or nonlinear relationship between cell dose and 12-month LVEF improvement (β = −0.44, p = 0.693; Figure 3B). Sensitivity analysis indicated that the pooled result was robust (Supplementary Figure S3B). Egger’s test did not suggest significant publication bias (p = 0.137; Supplementary Figure S4B).

#### Left ventricular ejection fraction at final follow-up

A total of 29 RCTs reported LVEF at final follow-up, including 943 patients in the BMMNC group and 762 patients in the control group. Random-effects meta-analysis showed that BMMNC therapy significantly improved LVEF at final follow-up compared with control treatment (MD = 2.24%, 95%CI: 0.77 to 3.71; I^2^ = 52.0%). The corresponding forest plot is shown in Figure 5.

**FIGURE 5:**
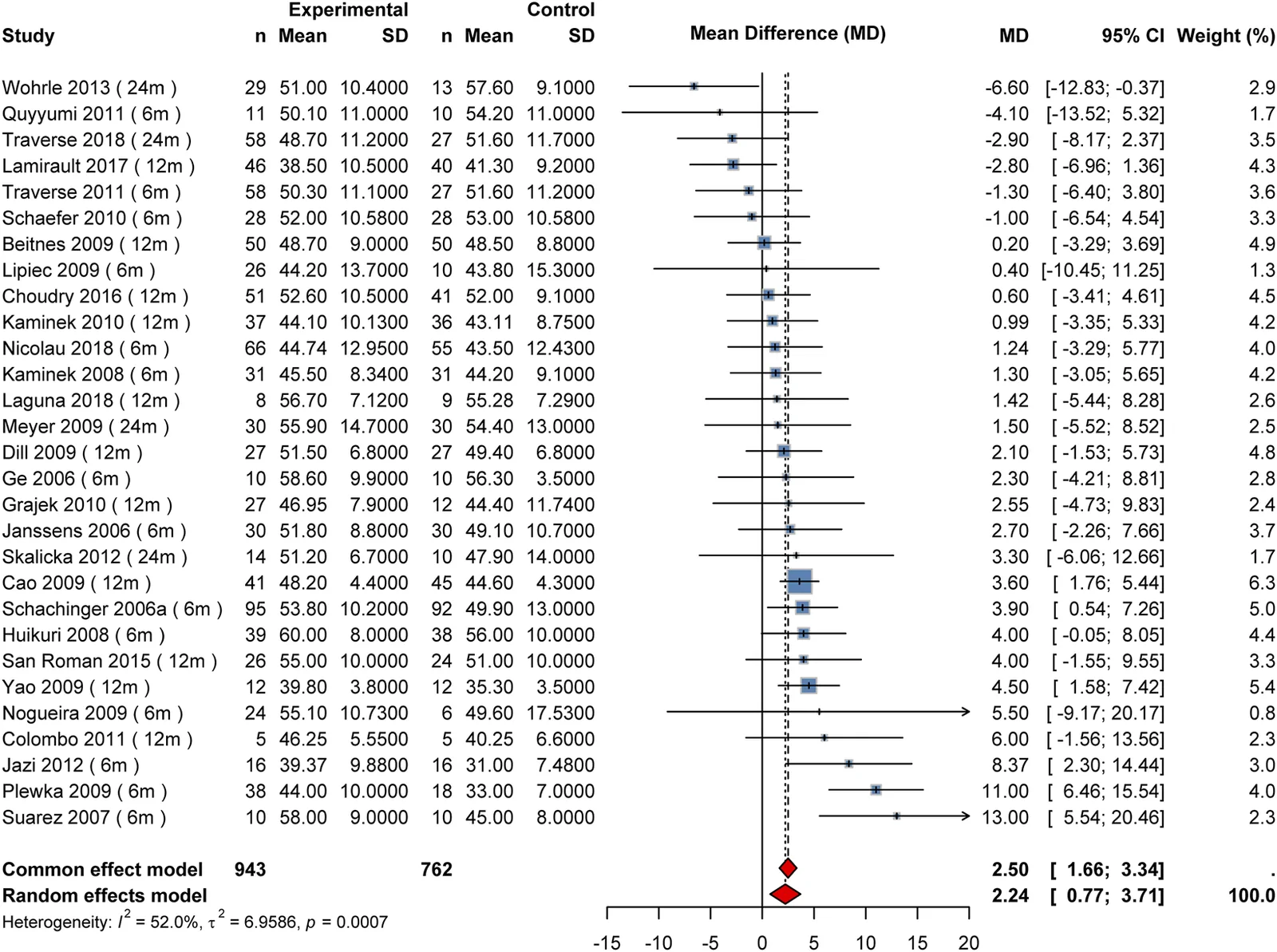
Forest plot of overall LVEF. Pooled mean difference in LVEF between BMMNC therapy and control groups across available follow-up time points.

Dose subgroup analysis showed that the intermediate-dose group (10^8^–10^9^ cells; 13 studies) showed a positive trend but did not reach statistical significance (MD = 2.38%, 95%CI: −0.03–4.78). The low-dose group (<10^8^ cells; 10 studies) and high-dose group (>10^9^ cells; six studies) also did not show statistically significant benefits. No significant between-subgroup difference was observed. The subgroup forest plot is shown in Supplementary Figure S2C.

Time subgroup analysis showed that the pooled effects at 6 months, 12 months, and 24 months were MD = 3.47% (95%CI: 0.85–6.09), MD = 2.09% (95%CI: 0.53–3.64), and MD = −1.97% (95%CI: −8.64 to 4.69), respectively (Supplementary Figure S2D). Meta-regression further confirmed that the LVEF benefit decreased with longer follow-up duration (β = −0.296% per month, 95%CI: −0.549 to −0.043; Supplementary Figure S5). Dose–response meta-regression did not identify a significant linear or nonlinear relationship between cell dose and LVEF improvement at final follow-up (β = 0.423, p = 0.607; Figure 3C). Sensitivity analysis indicated that the pooled result was robust (Supplementary Figure S3C). Egger’s test did not suggest significant publication bias (p = 0.502; Supplementary Figure S4C).

#### Major adverse cardiac events at final follow-up

A total of 32 RCTs reported MACE at final follow-up, including 1,794 patients in the BMMNC group and 1,387 patients in the control group. Random-effects meta-analysis showed that BMMNC therapy significantly reduced the risk of MACE at final follow-up compared with control treatment (RR = 0.62, 95%CI: 0.45 to 0.86; I^2^ = 0.0%). The corresponding forest plot is shown in Figure 6.

**FIGURE 6:**
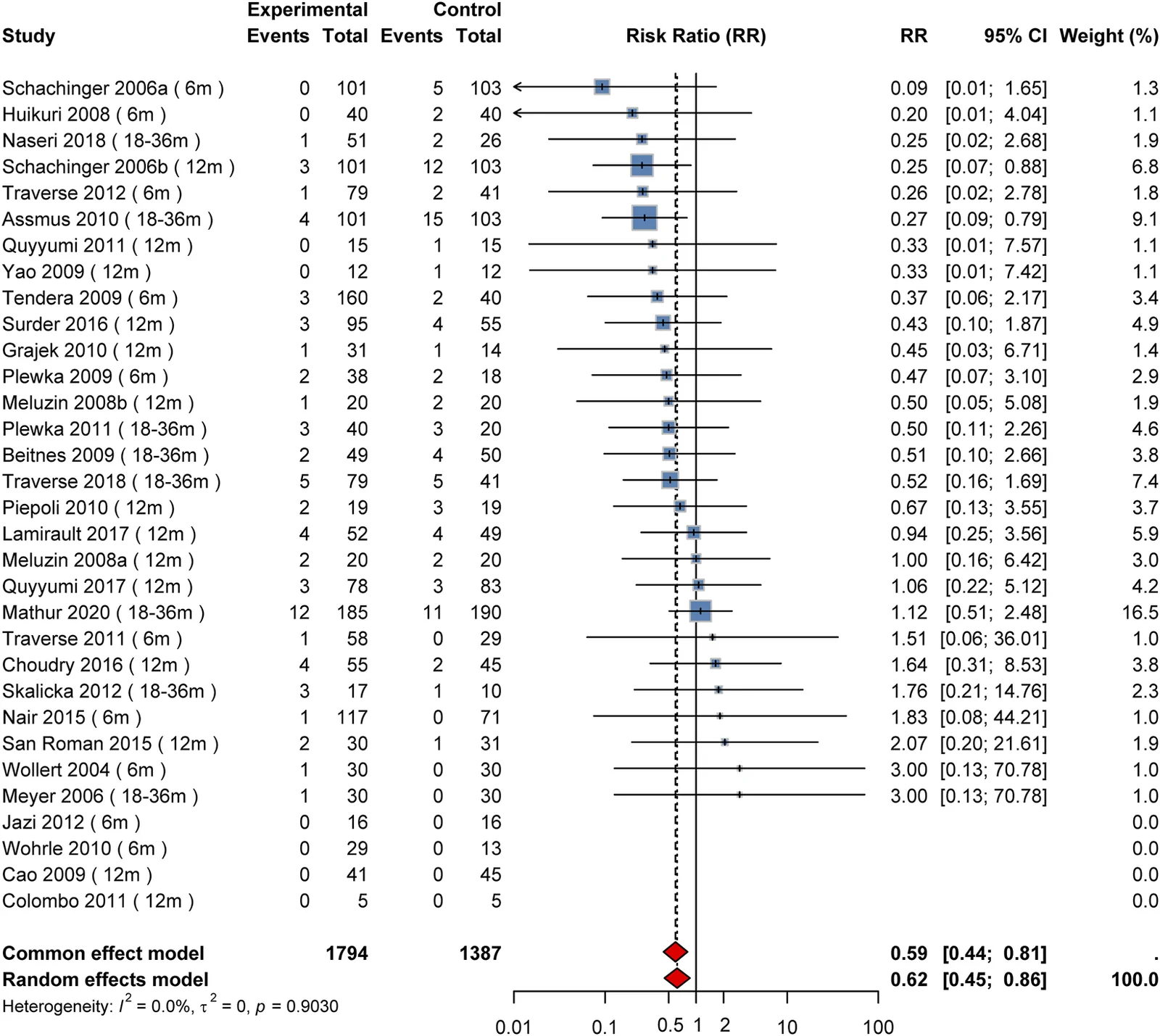
Forest plot of major adverse cardiac events. Pooled risk ratio for major adverse cardiac events (MACE) comparing BMMNC therapy with control treatment.

Dose subgroup analysis showed that the pooled RR was 0.58 (95%CI: 0.35–0.96) in the low-dose group (<10^8^ cells; 10 studies) and 0.59 (95%CI: 0.38–0.92) in the intermediate-dose group (10^8^–10^9^ cells; 17 studies), indicating that BMMNC therapy significantly reduced MACE risk in both groups. In contrast, the pooled RR in the high-dose group (>10^9^ cells; five studies) was 1.53 (95%CI: 0.40–5.82), showing no statistically significant benefit. No significant between-subgroup difference was observed. The subgroup forest plot is shown in Supplementary Figure S2E. Time subgroup analysis across 6 months, 12 months, and 18–36 months did not reveal significant differences between subgroups (Supplementary Figure S2F).

Dose–response meta-regression showed a significant positive association between log-transformed cell dose and MACE risk (β = 0.341 per log_10_ unit, 95%CI: 0.104 to 0.578; p = 0.006; Figure 3D). A mixed meta-regression model further confirmed that this dose effect was independent of follow-up duration (adjusted β = 0.347, p = 0.006; Supplementary Figures S6A, B), while follow-up duration itself was not significantly associated with MACE risk (p = 0.746). Sensitivity analysis indicated that the pooled result was robust (Supplementary Figure S3D). Egger’s test did not suggest significant publication bias (p = 0.966; Supplementary Figure S4D).

## Discussion

This study systematically evaluated RCT evidence on BMMNC therapy for MI and further examined its dose–response relationship and the influence of follow-up duration on treatment effects. The results showed that BMMNC therapy significantly improved LVEF at 6 months, and the overall final follow-up analysis also suggested a modest improvement in LVEF. However, the effect was attenuated at 12 months and no longer reached statistical significance. Meta-regression further indicated that the magnitude of LVEF improvement gradually decreased as follow-up duration increased. In parallel, BMMNC therapy was associated with an overall reduction in MACE risk. Dose–response analyses showed no clear linear or nonlinear association between cell dose and LVEF improvement, whereas higher cell doses were significantly associated with increased MACE risk. Taken together, these findings suggest that BMMNC therapy for MI may provide short-term cardiac functional benefit and overall clinical event reduction, but the durability of functional improvement appears limited, and high-dose therapy may carry potential safety concerns.

The observation that BMMNC therapy significantly improved LVEF at 6 months, with attenuation of this effect during longer follow-up, is biologically plausible. BMMNCs are a heterogeneous cell population, and their potential therapeutic effects are generally thought to depend primarily on paracrine signaling, inflammatory modulation, promotion of angiogenesis, and improvement of the local reparative microenvironment, rather than direct differentiation into cardiomyocytes and true myocardial regeneration. Therefore, their effects may be concentrated in the early repair phase after infarction. Potential mechanisms include attenuation of ischemia–reperfusion injury, improvement of microcirculation in the peri-infarct zone, and inhibition of early adverse ventricular remodeling, which may translate into short-term LVEF improvement (Rettkowski et al., 2025; Karim and Tompkins, 2025; Gnecchi et al., 2008; Prabhu and Frangogiannis, 2016; Yan et al., 2024). However, as infarct scar formation progresses and left ventricular remodeling becomes more established, a therapy mainly acting through microenvironmental modulation may be insufficient to reverse long-term structural damage (Rettkowski et al., 2025; Karim and Tompkins, 2025; Prabhu and Frangogiannis, 2016). This may explain why the 6-month results showed a clearer benefit, while the effect diminished or disappeared at 12 months and beyond.

Importantly, this study did not identify a significant linear or nonlinear dose–response relationship between cell dose and LVEF improvement. This finding suggests that the assumption that “a higher cell dose leads to greater efficacy” is not supported by the current RCT evidence for BMMNC therapy in MI. Several explanations are possible. First, BMMNCs are not a homogeneous cell product. Across studies, the cellular composition, progenitor cell proportion, cell viability, purity, and paracrine activity of the infused cell suspension may differ substantially. Therefore, total infused cell number may not accurately reflect the true biologically active dose (Yoon et al., 2010; Seeger et al., 2007; Van Beem et al., 2008). Second, the treatment effect of BMMNCs may also be influenced by baseline patient characteristics, infarct size, quality of reperfusion, timing of administration, route of delivery, and concomitant pharmacological therapy. Third, cell therapy may have a plateau effect, whereby increasing the number of infused cells beyond a certain threshold does not further enhance tissue repair (Mahram et al., 2025; Quyyumi et al., 2011; Schächinger et al., 2006; Traverse et al., 2012).

Compared with the LVEF findings, the safety and clinical event results provide another important observation: BMMNC therapy was associated with a significant overall reduction in MACE risk, with very low between-study heterogeneity. This suggests that, although the magnitude and durability of LVEF improvement may be limited, BMMNC therapy may still confer a net clinical benefit in terms of adverse event reduction. However, further meta-regression showed that increasing cell dose was associated with a significantly higher risk of MACE, and the high-dose subgroup did not show clear clinical benefit. The mechanisms by which high-dose BMMNC therapy may increase adverse event risk remain uncertain. One possible explanation is an increased local microcirculatory burden after cell infusion. In particular, with intracoronary infusion, a large number of cells may be retained within the microvascular bed, potentially leading to impaired microvascular perfusion or microembolization. In addition, high-dose heterogeneous cell populations may amplify local inflammatory responses or endothelial dysfunction, thereby exerting unfavorable effects on the vulnerable post-infarction myocardial microenvironment (Mahram et al., 2025; Gao et al., 2013; Fiarresga et al., 2015).

The subgroup findings suggest that the intermediate dose range of 10^8^ to 10^9^ cells may deserve particular attention. Within this range, LVEF improvement at 6 months reached statistical significance, the final follow-up effect showed a positive trend, and MACE risk was consistently reduced. In contrast, the low-dose group provided weaker evidence for cardiac functional improvement, while the high-dose group did not produce greater LVEF benefit and may have been associated with increased MACE risk. It should be emphasized that the dose categories in this study were based on discrete dose levels used in published trials rather than a prospectively designed standardized dose-escalation framework. Differences in cell-processing methods, cell viability, timing of infusion, and delivery route across studies mean that the same numerical dose of BMMNCs may not represent identical biological exposure (Lee et al., 2024; Mahram et al., 2025). Therefore, the dose range of 10^8^ to 10^9^ cells should be interpreted as a potentially reasonable range worthy of further validation rather than a definitive standard therapeutic dose.

The findings of this study are broadly consistent with previous research on BMMNC therapy for MI. Compared with earlier studies (Lee et al., 2024; Attar et al., 2022; Moeswir et al., 2025), the main added value of the present analysis is that it incorporated both follow-up duration and cell dose into a meta-regression framework. The results demonstrate a clear time-dependent attenuation of LVEF benefit and a dose-related increase in MACE risk. These findings may help explain discrepancies across previous RCTs and meta-analyses, and suggest that evaluation of BMMNC therapy should not focus solely on whether it is effective, but should also consider the durability of benefit and the dose range that offers the most favorable risk–benefit balance.

Several limitations should be acknowledged. First, the included RCTs spanned a long time period and differed in patient characteristics, MI type, reperfusion strategy, cell isolation and processing protocols, delivery route, timing of administration, and outcome assessment methods. These factors may have influenced the pooled estimates. Second, BMMNCs are a heterogeneous cell population, and most studies reported only the total cell number without detailed characterization of cellular composition, viability, or functional potency. Therefore, the dose variable in this study primarily reflects cell quantity and cannot be equated with the true biologically effective dose. In addition, dose units and reporting formats varied across studies; although attempts were made to standardize dose estimates, this process may have introduced additional uncertainty. Third, the dose–response analysis was based on study-level rather than individual patient-level data, and is therefore subject to ecological bias. The observed associations should be interpreted as between-study relationships rather than direct individual-level causal effects. Fourth, some subgroups, particularly the high-dose group, included a relatively small number of studies, which may limit statistical power and the ability to detect potential nonlinear dose–response patterns. Fifth, definitions of MACE were not fully consistent across studies. Although the overall direction of effect was stable, differences in endpoint composition may have affected the pooled analysis. Furthermore, only English-language publications were included, and language bias cannot be completely excluded. Finally, two additional limitations should be noted. Infarct size-related outcomes were not analyzed because they were reported in too few studies. In addition, route-specific effects could not be reliably evaluated because 42 of the 43 studies used intracoronary delivery, only one study used intramyocardial delivery, and no studies used intravenous delivery.

## Conclusions

BMMNC therapy may improve LVEF in the short term and reduce the overall risk of MACE in patients with MI, but the LVEF benefit appears to diminish with longer follow-up. Current evidence does not support a significant linear or nonlinear dose-response relationship between cell dose and LVEF improvement, although higher cell doses may be associated with increased MACE risk. Considering both efficacy and safety, 10^8^ to 10^9^ cells may represent a potentially favorable therapeutic dose range that warrants further validation. Future large-scale, high-quality RCTs with longer follow-up, standardized cell-processing protocols, and direct comparisons of delivery routes are needed to clarify the optimal dose, safety boundaries, ideal timing of administration, and patient populations most likely to benefit from BMMNC therapy, thereby more precisely defining its clinical role in regenerative treatment for MI.

## Data Availability

The original contributions presented in the study are included in the article/Supplementary Material, further inquiries can be directed to the corresponding author.
