# Supplementary material for: Efficacy and safety of bone marrow mononuclear cell therapy for myocardial infarction: a dose–response meta-analysis
Source: Front Cell Dev Biol. 2026 Oct 9;14:1952788. doi: 10.3389/fcell.2026.1952788 (PMC13690531; doi:10.3389/fcell.2026.1952788)
Supplement: Supplementary file 1 [file Supplementaryfile1.docx]

**Supplementary File S1.** Detailed search strategies for each database

**PubMed**

( "Myocardial Infarction"[Mesh] OR "myocardial infarction"[Title/Abstract] OR "myocardial infarctions"[Title/Abstract] OR "acute myocardial infarction"[Title/Abstract] OR "acute myocardial infarctions"[Title/Abstract] OR "AMI"[Title/Abstract] OR "STEMI"[Title/Abstract] OR "ST elevation myocardial infarction"[Title/Abstract] OR "ST-elevation myocardial infarction"[Title/Abstract] OR "ST segment elevation myocardial infarction"[Title/Abstract] OR "ST-segment elevation myocardial infarction"[Title/Abstract] OR "heart attack"[Title/Abstract] OR "heart attacks"[Title/Abstract] OR "cardiac infarction"[Title/Abstract] OR "cardiac infarctions"[Title/Abstract] OR "coronary infarction"[Title/Abstract] OR "coronary infarctions"[Title/Abstract] OR "myocardial infarct"[Title/Abstract] OR "myocardial infarcts"[Title/Abstract] OR "myocardial infarct*"[Title/Abstract] ) AND ( "Bone Marrow Cells"[Mesh] OR "Stem Cells"[Mesh] OR "Cell- and Tissue-Based Therapy"[Mesh] OR "bone marrow"[Title/Abstract] OR "bone marrow cell"[Title/Abstract] OR "bone marrow cells"[Title/Abstract] OR "bone marrow-derived cell"[Title/Abstract] OR "bone marrow-derived cells"[Title/Abstract] OR "bone marrow derived cell"[Title/Abstract] OR "bone marrow derived cells"[Title/Abstract] OR "bone marrow mononuclear cell"[Title/Abstract] OR "bone marrow mononuclear cells"[Title/Abstract] OR "mononuclear bone marrow cell"[Title/Abstract] OR "mononuclear bone marrow cells"[Title/Abstract] OR "bone marrow-derived mononuclear cell"[Title/Abstract] OR "bone marrow-derived mononuclear cells"[Title/Abstract] OR "bone marrow derived mononuclear cell"[Title/Abstract] OR "bone marrow derived mononuclear cells"[Title/Abstract] OR "BMMNC"[Title/Abstract] OR "BMMNCs"[Title/Abstract] OR "BM-MNC"[Title/Abstract] OR "BM-MNCs"[Title/Abstract] OR "BMC"[Title/Abstract] OR "BMCs"[Title/Abstract] OR "BMMC"[Title/Abstract] OR "BMMCs"[Title/Abstract] OR "bone marrow stem cell"[Title/Abstract] OR "bone marrow stem cells"[Title/Abstract] OR "bone marrow-derived stem cell"[Title/Abstract] OR "bone marrow-derived stem cells"[Title/Abstract] OR "bone marrow derived stem cell"[Title/Abstract] OR "bone marrow derived stem cells"[Title/Abstract] OR "bone marrow progenitor cell"[Title/Abstract] OR "bone marrow progenitor cells"[Title/Abstract] OR "bone marrow-derived progenitor cell"[Title/Abstract] OR "bone marrow-derived progenitor cells"[Title/Abstract] OR "bone marrow derived progenitor cell"[Title/Abstract] OR "bone marrow derived progenitor cells"[Title/Abstract] OR "autologous bone marrow cell"[Title/Abstract] OR "autologous bone marrow cells"[Title/Abstract] OR "autologous bone marrow-derived cell"[Title/Abstract] OR "autologous bone marrow-derived cells"[Title/Abstract] OR "intracoronary bone marrow cell"[Title/Abstract] OR "intracoronary bone marrow cells"[Title/Abstract] OR "intramyocardial bone marrow cell"[Title/Abstract] OR "intramyocardial bone marrow cells"[Title/Abstract] OR "cell therapy"[Title/Abstract] OR "cell transplantation"[Title/Abstract] OR "stem cell therapy"[Title/Abstract] OR "stem cell transplantation"[Title/Abstract] )

**Embase**

('myocardial infarction'/exp OR 'myocardial infarction':ti,ab,kw OR 'myocardial infarctions':ti,ab,kw OR 'acute myocardial infarction':ti,ab,kw OR 'acute myocardial infarctions':ti,ab,kw OR ami:ti,ab,kw OR stemi:ti,ab,kw OR 'st elevation myocardial infarction':ti,ab,kw OR 'st-elevation myocardial infarction':ti,ab,kw OR 'st segment elevation myocardial infarction':ti,ab,kw OR 'st-segment elevation myocardial infarction':ti,ab,kw OR 'heart attack':ti,ab,kw OR 'heart attacks':ti,ab,kw OR 'cardiac infarction':ti,ab,kw OR 'cardiac infarctions':ti,ab,kw OR 'coronary infarction':ti,ab,kw OR 'coronary infarctions':ti,ab,kw OR 'myocardial infarct':ti,ab,kw OR 'myocardial infarcts':ti,ab,kw OR 'myocardial infarct*':ti,ab,kw) AND ('bone marrow cell'/exp OR 'stem cell'/exp OR 'cell therapy'/exp OR 'bone marrow':ti,ab,kw OR 'bone marrow cell':ti,ab,kw OR 'bone marrow cells':ti,ab,kw OR 'bone marrow-derived cell':ti,ab,kw OR 'bone marrow-derived cells':ti,ab,kw OR 'bone marrow derived cell':ti,ab,kw OR 'bone marrow derived cells':ti,ab,kw OR 'bone marrow mononuclear cell':ti,ab,kw OR 'bone marrow mononuclear cells':ti,ab,kw OR 'mononuclear bone marrow cell':ti,ab,kw OR 'mononuclear bone marrow cells':ti,ab,kw OR 'bone marrow-derived mononuclear cell':ti,ab,kw OR 'bone marrow-derived mononuclear cells':ti,ab,kw OR 'bone marrow derived mononuclear cell':ti,ab,kw OR 'bone marrow derived mononuclear cells':ti,ab,kw OR bmmnc:ti,ab,kw OR bmmncs:ti,ab,kw OR 'bm-mnc':ti,ab,kw OR 'bm-mncs':ti,ab,kw OR bmc:ti,ab,kw OR bmcs:ti,ab,kw OR bmmc:ti,ab,kw OR bmmcs:ti,ab,kw OR 'bone marrow stem cell':ti,ab,kw OR 'bone marrow stem cells':ti,ab,kw OR 'bone marrow-derived stem cell':ti,ab,kw OR 'bone marrow-derived stem cells':ti,ab,kw OR 'bone marrow derived stem cell':ti,ab,kw OR 'bone marrow derived stem cells':ti,ab,kw OR 'bone marrow progenitor cell':ti,ab,kw OR 'bone marrow progenitor cells':ti,ab,kw OR 'bone marrow-derived progenitor cell':ti,ab,kw OR 'bone marrow-derived progenitor cells':ti,ab,kw OR 'bone marrow derived progenitor cell':ti,ab,kw OR 'bone marrow derived progenitor cells':ti,ab,kw OR 'autologous bone marrow cell':ti,ab,kw OR 'autologous bone marrow cells':ti,ab,kw OR 'autologous bone marrow-derived cell':ti,ab,kw OR 'autologous bone marrow-derived cells':ti,ab,kw OR 'intracoronary bone marrow cell':ti,ab,kw OR 'intracoronary bone marrow cells':ti,ab,kw OR 'intramyocardial bone marrow cell':ti,ab,kw OR 'intramyocardial bone marrow cells':ti,ab,kw OR 'cell therapy':ti,ab,kw OR 'cell transplantation':ti,ab,kw OR 'stem cell therapy':ti,ab,kw OR 'stem cell transplantation':ti,ab,kw)

**Cochrane Library**

#1 MeSH descriptor: [Myocardial Infarction] explode all trees

#2 ("myocardial infarction" OR "myocardial infarctions" OR "acute myocardial infarction" OR "acute myocardial infarctions" OR AMI OR STEMI OR "ST elevation myocardial infarction" OR "ST-elevation myocardial infarction" OR "ST segment elevation myocardial infarction" OR "ST-segment elevation myocardial infarction" OR "heart attack" OR "heart attacks" OR "cardiac infarction" OR "cardiac infarctions" OR "coronary infarction" OR "coronary infarctions" OR "myocardial infarct" OR "myocardial infarcts" OR (myocardial NEXT infarct*)):ti,ab,kw

#3 #1 OR #2

#4 MeSH descriptor: [Bone Marrow Cells] explode all trees

#5 MeSH descriptor: [Stem Cells] explode all trees

#6 MeSH descriptor: [Cell- and Tissue-Based Therapy] explode all trees

#7 ("bone marrow" OR "bone marrow cell" OR "bone marrow cells" OR "bone marrow-derived cell" OR "bone marrow-derived cells" OR "bone marrow derived cell" OR "bone marrow derived cells" OR "bone marrow mononuclear cell" OR "bone marrow mononuclear cells" OR "mononuclear bone marrow cell" OR "mononuclear bone marrow cells" OR "bone marrow-derived mononuclear cell" OR "bone marrow-derived mononuclear cells" OR "bone marrow derived mononuclear cell" OR "bone marrow derived mononuclear cells" OR BMMNC OR BMMNCs OR BM-MNC OR BM-MNCs OR BMC OR BMCs OR BMMC OR BMMCs OR "bone marrow stem cell" OR "bone marrow stem cells" OR "bone marrow-derived stem cell" OR "bone marrow-derived stem cells" OR "bone marrow derived stem cell" OR "bone marrow derived stem cells" OR "bone marrow progenitor cell" OR "bone marrow progenitor cells" OR "bone marrow-derived progenitor cell" OR "bone marrow-derived progenitor cells" OR "bone marrow derived progenitor cell" OR "bone marrow derived progenitor cells" OR "autologous bone marrow cell" OR "autologous bone marrow cells" OR "autologous bone marrow-derived cell" OR "autologous bone marrow-derived cells" OR "intracoronary bone marrow cell" OR "intracoronary bone marrow cells" OR "intramyocardial bone marrow cell" OR "intramyocardial bone marrow cells" OR "cell therapy" OR "cell transplantation" OR "stem cell therapy" OR "stem cell transplantation"):ti,ab,kw

#8 #4 OR #5 OR #6 OR #7

#9 #3 AND #8

**Web of Science**

TS=("myocardial infarction" OR "myocardial infarctions" OR "acute myocardial infarction" OR "acute myocardial infarctions" OR "AMI" OR "STEMI" OR "ST elevation myocardial infarction" OR "ST-elevation myocardial infarction" OR "ST segment elevation myocardial infarction" OR "ST-segment elevation myocardial infarction" OR "heart attack" OR "heart attacks" OR "cardiac infarction" OR "cardiac infarctions" OR "coronary infarction" OR "coronary infarctions" OR "myocardial infarct" OR "myocardial infarcts" OR "myocardial infarct*") AND TS=("bone marrow" OR "bone marrow cell" OR "bone marrow cells" OR "bone marrow-derived cell" OR "bone marrow-derived cells" OR "bone marrow derived cell" OR "bone marrow derived cells" OR "bone marrow mononuclear cell" OR "bone marrow mononuclear cells" OR "mononuclear bone marrow cell" OR "mononuclear bone marrow cells" OR "bone marrow-derived mononuclear cell" OR "bone marrow-derived mononuclear cells" OR "bone marrow derived mononuclear cell" OR "bone marrow derived mononuclear cells" OR "BMMNC" OR "BMMNCs" OR "BM-MNC" OR "BM-MNCs" OR "BMC" OR "BMCs" OR "BMMC" OR "BMMCs" OR "bone marrow stem cell" OR "bone marrow stem cells" OR "bone marrow-derived stem cell" OR "bone marrow-derived stem cells" OR "bone marrow derived stem cell" OR "bone marrow derived stem cells" OR "bone marrow progenitor cell" OR "bone marrow progenitor cells" OR "bone marrow-derived progenitor cell" OR "bone marrow-derived progenitor cells" OR "bone marrow derived progenitor cell" OR "bone marrow derived progenitor cells" OR "autologous bone marrow cell" OR "autologous bone marrow cells" OR "autologous bone marrow-derived cell" OR "autologous bone marrow-derived cells" OR "intracoronary bone marrow cell" OR "intracoronary bone marrow cells" OR "intramyocardial bone marrow cell" OR "intramyocardial bone marrow cells" OR "cell therapy" OR "cell transplantation" OR "stem cell therapy" OR "stem cell transplantation")

**Scopus**

TITLE-ABS-KEY( ( "myocardial infarction" OR "myocardial infarctions" OR "acute myocardial infarction" OR "acute myocardial infarctions" OR AMI OR STEMI OR "ST elevation myocardial infarction" OR "ST-elevation myocardial infarction" OR "ST segment elevation myocardial infarction" OR "ST-segment elevation myocardial infarction" OR "heart attack" OR "heart attacks" OR "cardiac infarction" OR "cardiac infarctions" OR "coronary infarction" OR "coronary infarctions" OR "myocardial infarct" OR "myocardial infarcts" OR "myocardial infarct*" ) AND ( "bone marrow" OR "bone marrow cell" OR "bone marrow cells" OR "bone marrow-derived cell" OR "bone marrow-derived cells" OR "bone marrow derived cell" OR "bone marrow derived cells" OR "bone marrow mononuclear cell" OR "bone marrow mononuclear cells" OR "mononuclear bone marrow cell" OR "mononuclear bone marrow cells" OR "bone marrow-derived mononuclear cell" OR "bone marrow-derived mononuclear cells" OR "bone marrow derived mononuclear cell" OR "bone marrow derived mononuclear cells" OR BMMNC OR BMMNCs OR BM-MNC OR BM-MNCs OR BMC OR BMCs OR BMMC OR BMMCs OR "bone marrow stem cell" OR "bone marrow stem cells" OR "bone marrow-derived stem cell" OR "bone marrow-derived stem cells" OR "bone marrow derived stem cell" OR "bone marrow derived stem cells" OR "bone marrow progenitor cell" OR "bone marrow progenitor cells" OR "bone marrow-derived progenitor cell" OR "bone marrow-derived progenitor cells" OR "bone marrow derived progenitor cell" OR "bone marrow derived progenitor cells" OR "autologous bone marrow cell" OR "autologous bone marrow cells" OR "autologous bone marrow-derived cell" OR "autologous bone marrow-derived cells" OR "intracoronary bone marrow cell" OR "intracoronary bone marrow cells" OR "intramyocardial bone marrow cell" OR "intramyocardial bone marrow cells" OR "cell therapy" OR "cell transplantation" OR "stem cell therapy" OR "stem cell transplantation" ))

**Supplementary Figure S1.** Detailed results of the risk-of-bias assessment.

**Supplementary Figure S2A.** Dose subgroup forest plot for 6-month LVEF

**Supplementary Figure S3A.** Leave-one-out sensitivity analysis for 6-month LVEF

**Supplementary Figure S4A.** Funnel plot for publication bias of LVEF at 6 months

**Supplementary Figure S2B.** Dose subgroup forest plot for 12-month LVEF

**Supplementary Figure S3B.** Leave-one-out sensitivity analysis for 12-month LVEF

**Supplementary Figure S4B.** Funnel plot for 12-month LVEF

**Supplementary Figure S2C.** Dose subgroup forest plot for endpoint LVEF

**Supplementary Figure S2D.** Time subgroup forest plot for endpoint LVEF

**Supplementary Figure S3C.** Leave-one-out sensitivity analysis for endpoint LVEF

 **Supplementary Figure S4C.** Funnel plot for endpoint LVEF

 **Supplementary Figure S5.** Time-effect meta-regression for endpoint LVEF

**Supplementary Figure S2E.** Dose subgroup forest plot for endpoint MACE

**Supplementary Figure S2F.** Time subgroup forest plot for endpoint MACE

 **Supplementary Figure S3D.** Leave-one-out sensitivity analysis for endpoint MACE

**Supplementary Figure S4D.** Funnel plot for endpoint MACE

**Supplementary Figure S6A.** Mixed meta-regression contour plot for endpoint MACE

**Supplementary Figure S6B.** Mixed meta-regression by follow-up time for endpoint MACE
